# Use of medicinal plants during COVID-19 pandemic in Brazil

**DOI:** 10.1038/s41598-023-43673-y

**Published:** 2023-10-02

**Authors:** Alciellen Mendes da Silva, Ana Luísa Horsth, Élida da Silva Timóteo, Ronaldo José Faria, Patrícia Silva Bazoni, Eduardo Frizzera Meira, Jéssica Barreto Ribeiro dos Santos, Michael Ruberson Ribeiro da Silva

**Affiliations:** 1 Health Evaluation, Technology, and Economics Group, Federal University of Espírito Santo, Alegre, 29500-000 Brazil; 2 Graduate Program of Pharmaceutical Services, Federal University of Espírito Santo, Alegre, 29500-000 Brazil

**Keywords:** Epidemiology, Public health

## Abstract

Medicinal plants are an integrative and complementary health practice widely used by the population. However, its use is not without risks. This study assessed the profile and associated factors with the traditional use of medicinal plants. To this end, a cross-sectional survey study was conducted in a southeastern Brazilian city. Descriptive analysis was performed by frequency distribution and median and interquartile range. Associated factors with the use of medicinal plants were analyzed using Poisson regression with robust variance. A total of 641 people were interviewed, of whom 258 (40.2%) reported using medicinal plants. A total of 79 distinct plants were identified, of whom *Melissa officinalis* (31.0%), *Peumus boldus* (24.4%), *Mentha spicata* (20.9%), *Matricaria recutita* L. (18.2%), *Rosmarinus officinalis* (17.0%), and *Foeniculum vulgare* (14.7%) were the most used. There were no reports of medicinal plants used to treat COVID-19. However, anxiety was the most frequently cited indication for using medicinal plants, a health condition exacerbated by the COVID-19 pandemic. Furthermore, the use of medicinal plants for treating respiratory and gastrointestinal symptoms similar to those in COVID-19 has been identified. There was an association between the use of medicinal plants and females, non-white, lower schooling, higher income, and comorbidities.

## Introduction

The use of medicinal plants by the population as an alternative therapy to treat many diseases has been an old common practice over the years^1^. All the traditional knowledge about medicinal plants passed down from generation to generation has contributed to people’s health, especially in rural or small communities that live in remote places without adequate health infrastructure^2,3^.

Medicinal plants generate health benefits when used rationally and adequately. They contain a large arsenal of chemical components that can be useful but also represent a possible health risk. Thus, users, healthcare providers, and prescribers must know about the plant, including proper identification, conservation, method of preparation and use, and any potential side effects^4^. There is a long history of using medicinal plants in Brazil due to its rich biodiversity and cultural heritage^1,2^. In this regard, the National Program of Medicinal Plants and Herbal Medicines was established in 2016 to provide safe and free access to medicinal plants in Brazilian primary healthcare^4^.

Recent studies have reported the use of medicinal plants for the prevention and treatment of COVID-19 in different countries around the world, such as Peru, Thailand, and Nepal. However, studies to validate the use of these plants for COVID-19 are needed, as additional care is required to avoid health issues associated with their use^5,6^.

In this regard, the present study aimed to identify the most commonly used medicinal plants and understand the consumer profile and the factors associated with their use during the COVID-19 pandemic in Brazil. As a result, it will be possible to contribute to appropriately informing the population about the benefits, risks, and safe use of widely used medicinal plants, contributing to people’s quality of life and preserving a long-standing tradition in Brazil and worldwide.

## Results

### Sociodemographic and clinical characteristics

A total of 641 people were interviewed. Most were women (72.9%), Caucasian (47.3%), married (43.7%), Catholic (49.1%), and had only completed primary school (57.4%). Furthermore, 46.8% of the population earned a minimum wage (Table 1).

**Table 1:** Sociodemographic characteristics of the study population.

Variables	Use plants	Do not use plants	Total	p-value
Age in years, mean (SD)	56.51 (16.92)	51.01 (19.64)	53.09 (18.83)	< 0.001
Sex				
Female (n, %)	217 (43.9)	277 (56.1)	494 (72.9)	< 0.001
Male (n, %)	52 (28.3)	132 (71.7)	184 (27.1)	
Race				0.010
White/caucasian (n, %)	130 (40.6)	190 (59.4)	320 (47.3)	
Brown (n, %)	76 (33.0)	154 (67.0)	230 (34.0)	
Others (n, %)	62 (49.2)	64 (50, 8)	126 (18.6)	
Region				0.992
Districts	81 (39.7)	123 (60.3)	204 (30.1)	
Municipality seat	188 (39.7)	285 (60.3)	473 (69.9)	
Marital status				0.042
Single (n, %)	56 (31.6)	121 (68.4)	177 (26.1)	
Married (n, %)	126 (42.6)	170 (57.4)	296 (43.7)	
Others (n, %)	86 (42.2)	118 (57.8)	204 (30.1)	
Religion				0.212
No religion (n, %)	15 (29.4)	36 (70.6)	51 (7.5)	
Catholic (n, %)	128 (38.4)	205 (61.6)	333 (49.1)	
Evangelical (n, %)	104 (38.7)	145 (35.5)	249 (36.7)	
Others (n, %)	22 (48.9)	23 (5.6)	45 (6.6)	
Schooling				0.032
Up to Complete Elementary	221 (56.7)	169 (43.3)	390 (57.4)	
Higher School Complete or more	188 (64.8)	102 (37.2)	85 (42.6)	
Income				0.110
≤ 1 minimun wage	111 (37.5)	188 (62.5)	299 (46.8)	
1–2 minimum wages	111 (40.5)	163 (59.5)	274 (42.9)	
> 2 minimum wages	34 (51.5)	32 (48.5)	66 (10.3)	

We observed statistically significant differences in age, sex, race, and marital status, with a higher proportion of women, self-declared Black (others), and married people using plants. Furthermore, medicinal plants were more commonly used by older adults (p-value < 0.05) (Table 1).

Regarding the clinical characteristics of the respondents, more than 50% reported good or very good health, while 40.1% reported regular health. Nearly 50% slept for more than 7 h per night, 80% had medical appointments over the past year, 65% did not engage in physical activity, 85.6% did not smoke, and 74.9% did not use alcohol. Furthermore, 77.6% had no private health insurance, 20.5% used five or more drugs (polypharmacy), 70% self-medicated, and 45.4% had two or more diseases. Hypertension, anxiety, dyslipidemia, and depression were the most common diseases (Table 2).

**Table 2:** Clinical characteristics of the study population.

Variables	Use plants	Do not use plants	Total	p-value
Health self-perception				
Very good or good	124 (45.8)	228 (55.7)	352 (51.8)	0.023
Regular	119 (43.9)	154 (37.7)	273 (40.1)	
Bad or Very Bad	28 (10.3)	27 (6.6)	55 (8.1)	
Quality of life	0.843 (0.171)	0.872 (0.176)	0.862 (0.173)	0.033
Body Mass Index	27.57 (5.90)	26.75 (5.46)	26.08 (5.67)	0.074
Medical consultations in the last year				0.240
Yes	221 (81.9)	318 (78.1)	539 (79.6)	
No	49 (18.1)	89 (21.9)	138 (20.4)	
Physical activity				0.764
Yes	94 (34.8)	149 (35.9)	241 (35.5)	
No	176 (65.2)	262 (64.1)	438 (64.5)	
Sleep				0.511
< 6 h	69 (25.7)	88 (21.6)	157 (23.2)	
From 6 to 7 h	73 (27.1)	112 (27.5)	185 (27.3)	
From 7 to 8 h	88 (32.7)	135 (33.1)	223 (32.9)	
< 8 h	39 (14.5)	73 (17.9)	112 (16.5)	
Smoke				0.517
Yes	39 (14.4)	52 (12.7)	91 (13.4)	
Nos	231 (85.6)	357 (87.3)	588 (86.6)	
Alcohol consumption				0.422
Never	210 (78.1)	298 (72.9)	508 (74.9)	
Daily	7 (2.6)	13 (3.2)	20 (2.9)	
Weekly	25 (9.3)	53 (13.0)	78 (11.5)	
Monthly	27 (10.0)	45 (11.0)	72 (10.6)	
Private health plan				0.163
Yes	68 (25.1)	84 (20.5)	152 (22.4)	
No	203 (74.9)	325 (79.5)	528 (77.6)	
Polypharmacy (5 or more drugs)				0.346
Yes	60 (22.3)	79 (19.3)	139 (20.5)	
No	209 (77.7)	330 (80.7)	539 (79.5)	
Self-medication				0.579
Yes	183 (70.4)	261 (68.3)	444 (69.2)	
No	77 (29.6)	121 (31.7)	198 (30.8)	
Number of diseases				
0 or 1	64 (23.6)	186 (45.5)	250 (36.8)	< 0.001
Two or more	207 (76.4)	223 (54.5)	430 (63.2)	
Main comorbidities				
Hypertension	148 (54.6)	164 (40.1)	312 (45.9)	< 0.001
Anxiety	142 (52.4)	162 (39.6)	304 (44.7)	0.001
Dyslipidemia	75 (27.7)	94 (23.0)	169 (24.9)	0.166
Depression	73 (26.9)	62 (15.2)	135 (19.9)	< 0.001
Obesity	51 (18.8)	59 (14.4)	110 (16.2)	0.128
Arthritis	45 (16.6)	58 (14.2)	103 (15.1)	0.388
Diabetes	45 (16.6)	52 (12.7)	97 (14.3)	0.155
Gastroesophageal reflux	57 (21.0)	37 (9.0)	94 (13.8)	< 0.001
Kidney diseases	39 (14.4)	35 (8.6)	74 (10.9)	0.017
Heart disease	28 (10.4)	45 (11.0)	73 (10.8)	0.795
Hypothyroidism	24 (8.9)	30 (7.4)	54 (8.0)	0.478
Asthma	20 (7.4)	23 (5.6)	43 (6.3)	0.357
Cancer	13 (4.8)	9 (2.2)	22 (3.2)	0.061

The two groups differed statistically concerning self-perception of health, quality of life, and the number of diseases, and the group using plants had a lower proportion of people reporting a very good or good self-perception of health, a lower quality of life, and a higher percentage of people had more than two diseases (p < 0.05) (Table 2).

### Use of medicinal plants

A total of 258 out of 641 participants (40.2%) reported using medicinal plants, and 79 distinct species were identified. The prominent families of plants found in use in the population were Asteraceae (n = 9), Lamiaceae (n = 8), Zingiberaceae (n = 4), Fabaceae (n = 4), Myrtaceae (n = 3), Cucurbitaceae (n = 3), Poaceae (n = 3), Apiaceae (n = 3), Rutaceae (n = 3), Malvaceae (n = 2), Rubiaceae (n = 2), Lauraceae (n = 2) and Anacardiaceae (n = 2). The other families had only one plant in use reported (Table 3).

**Table 3:** Medicinal plants reported by the study population.

Scientific name	Family	Scientific name	Family
Achyrocline satureioides	Asteraceae	*Kalanchoe brasiliensis Cambess*	Crassulaceae
*Bidens alba*	Asteraceae	*Leonurus sibiricus* L.	Lamiaceae
*Allium sativum* L.	Amaryllidaceae	*Lepidium meyenii*	Brassicaceae
*Aloe vera*	Xanthorrhoeaceae	*Lippia alba*	Verbenaceae
*Alpinia zerumbet*	Zingiberaceae	*Matricaria recutita* L.	Asteraceae
*Anadenanthera macrocarpa*	Fabaceae	*Maytenus ilicifolia*	Celastraceae
*Ananas comosus*	Bromeliaceae	*Melissa officinalis* L.	Lamiaceae
*Annona muricata*	Annonaceae	*Mentha pulegium* L.	Lamiaceae
*Apis mellifera* L.	Apoidea	*Mentha viridis*	Lamiaceae
*Artemisia absinthium* L.	Asteraceae	*Mentha spicata* L.	Lamiaceae
*Baccharis trimera*	Asteraceae	*Mikania glomerata*	Asteraceae
*Butia eriospatha*	Arecaceae	*Morinda citrifolia* L.	Rubiaceae
*Cajanus cajan*	Fabaceae	*Morus nigra* L.	Moraceae
*Camellia sinensis*	Theaceae	*Ocimum basilicum* L.	Lamiaceae
*Carica papaya* L.	Caricaceae	*Olea europaea* L.	Oliaceae
*Cecropia*	Urticaceae	*Paullinia cupana*	Sapindaceae
*Cinnamomum verum*	Lauraceae	*Passiflora edulis*	Passifloraceae
*Citrus limonum* L.	Rutaceae	*Pereskia aculeata*	Cactaceae
*Citrus reticulata* L.	Rutaceae	*Persea americana*	Lauraceae
*Citrus sinensis* L.	Rutaceae	*Petroselinum crispum*	Apiaceae
*Coffea *sp.	Rubiaceae	*Peumus boldus Molina*	Monimiaceae
*Cordia verbenacea* DC	Boraginaceae	*Pimpinella anisum* L.	Apiaceae
*Costus spicatus Swartz*	Zingiberaceae	*Plantago major* L.	Plantaginaceae
*Cucumis melo* L.	Cucurbitaceae	*Poa annua* L.	Poaceae
*Cucumis sativus* L.	Cucurbitaceae	*Psidium guajava *L.	Myrtaceae
*Cuphea carthagenensis*	Lythraceae	*Quassia amara* L.	Simaroubaceae
*Cymbopogon citratus*	Poaceae	*Rosa alba* L.	Rosaceae
*Dysphania ambrosioides*	Amaranthaceae	*Rosmarinus officinalis* L.	Lamiaceae
*Echinodorus grandiflorus*	Alismataceae	*Salvia officinalis* L.	Lamiaceae
*Equisetum arvense* L.	Equisetaceae	*Schinus terebinthifolius*	Anacardiaceae
*Erythrina verna*	Fabaceae	*Sechium edule*	Cucurbitaceae
*Erythroxylum vacciniifolium*	Erythroxylaceae	*Senna alexandrina*	Fabaceae
*Eugenia uniflora* L.	Myrtaceae	*Solanum cernuum*	Solanaceae
*Gossypium herbaceum* L.	Malvaceae	*Solidago chilensis*	Asteraceae
*Foeniculum vulgare*	Apiaceae	*Syzygium aromaticum*	Myrtaceae
*Hibiscus rosa-sinensis*	Malvaceae	*Taraxacum officinale* L.	Asteraceae
*Hypericum perforatum* L.	Hypericaceae	*Curcuma longa* L.	Zingiberaceae
*Ilex paraguariensis*	Aquifoliaceae	*Vernonia polyanthes*	Asteraceae
*Imperata brasiliensis*	Poaceae	*Zingiber officinale*	Zingiberaceae
*Mangifera indica* L.	Anacardiaceae		

*Melissa officinalis* (n = 80; 31.0%), *Peumus boldus* (n = 63; 24.4%), *Mentha spicata* (n = 54; 20.9%), *Matricaria recutita *L. (n = 47; 18.2%), *Rosmarinus officinalis* (n = 44; 17.0%), *Foeniculum vulgare* (n = 38; 14.7%), *Leonurus sibiricus *L. (n = 26; 10.0%), *Citrus limonum *L. (n = 25; 9.7%), and *Plantago major* (n = 16; 6.2%) were the most frequently utilized plants (Supplementary Material 1).

*Melissa officinalis*, *P. boldus*, *M. spicata*, *M. recutita* L., *R. officinalis* were the most common plants indicated for anxiety, and *F. vulgare*. *P. boldus,* and L*. sibiricus* L*.* were used for stomach issues, whereas *P. major* and *C. limonum* L. were more commonly utilized for flu. Plants used to prevent or treat COVID-19 have not been reported (Supplementary Material 1).

### Factors associated with medicinal plant use

Sex, race, schooling, wage, and diseases were associated with medicinal plant use. Women were 46% more likely than men to use medicinal plants, while people who self-identified as Black or other race (yellow or Indigenous) were 30% more likely than white people to use medicinal plants. Furthermore, those who had completed primary school had a 28% higher prevalence of using plants than people who had completed high school or higher. Those earning more than two minimum wages had a 61% higher prevalence of using medicinal plants than respondents earning up to two minimum wages. People with two or more diseases had a 59% higher prevalence of using medicinal plants than those with none or one disease (Table 4).

**Table 4:** Multivariate analysis of associated factors to medicinal plant use.

Variables	PR	CI 95%	p-value
Sex			
Male	1.00		
Female	1.46	1.13–1.89	0.004
Race			
White	1.00		
Brown	0.85	0.68–1.07	0.175
Black/Other	1.30	1.04–1.62	0.020
Schooling			
High School or more	1.00		
Up to Complete Elementary	1.28	1.04–1.57	0.020
Income			
Up to 1 minimum wage	1.00		
1–2 minimum wage	1.04	0.85–1.27	0.737
> 2 minimum wages	1.61	1.20–2.16	0.002
Disease			
0 or 1	1.00		
2 or more	1.59	1.24–2.04	< 0.001

PR, prevalence ratio; CI, confidence interval.

## Discussion

The present leading study looked at medicinal plant use during COVID-19 in southeastern Brazil. No other similar studies were found in Brazil during the pandemic period. In this regard, our findings were compared to other national studies conducted before the COVID-19 pandemic and international studies conducted during the COVID-19 pandemic.

We discovered that 40.2% of the study population used medicinal plants, comparable to the 42.7% found by Gonçalves et al.^7^ in a small northeastern Brazilian city. However, Araujo et al.^8^ discovered that 79% of respondents treated at a primary health unit in northeastern Brazil used medicinal plants, which is higher than the percentage found in our study. The higher prevalence found by Araújo et al.^8^ could be attributed to the fact that it measured the use of medicinal plants in individuals who were followed up in primary health care, as opposed to the current study, which estimated the use of plant medicinal products through a population-based study. Conversely, medicinal plants were utilized by only 21% of patients receiving treatment at primary health centers in southern Brazil^9^. As a result, we identified that the prevalence of the use of medicinal plants varies greatly across Brazil. The northernmost municipalities have the highest use and the southernmost municipalities having the lowest use^8,9^.

A total of 79 medicinal plants were used by the population, and the most common ones were *M. officinalis* (31.0%), *P. boldus* (24.4%), *M. spicata* (20.9%), *M. recutita* L. (18.2%), *Rosmarinus officinalis* (17.0%), *F. vulgare* (14.7%), *L. sibiricus* L. (10.0%), *C. limonum* L. (9.7%), and *P. major* (6.2%). According to Araujo et al. (2014), the most common plants used were *P. boldus* (21.0%), *M. officinalis* (14.3%), *Cymbopogon citratus* (7.7%), *Coleus amboinicus* (6.7%), *M. recutita* L. (5.4%), and *F. vulgare* (5.3%), indicating similarities with the most common medicinal plants used in the current study. Zeni et al.^9^ also found similarities in the medicinal plants most used in Blumenau, which were *M. officinalis* (17.3%), *M. recutita* L. (12.6%), *M. spicata* (9.3%), *C. limonum* L. (7.3%), and *P. boldus* (6.6%)^8^. Therefore, based on different studies, the COVID-19 pandemic did not affect the consumption profile of medicinal plants in Brazil^7,9^.

There were no reports of medicinal plants used explicitly to treat or prevent COVID-19 by users. Anxiety was the most common reason for using *M. officinalis, M. spicata, M. recutita* L.*, R. officinalis,* and *F. vulgare.* This is especially important given how the COVID-19 pandemic has exacerbated anxiety and depression symptoms worldwide. According to recent data, the pandemic increased the prevalence of anxiety in the population by 25.6%, which was the primary indication for the most commonly used medicinal plants in the current study^10^.

*P. boldus* and *Leonurus sibiricus* L. were used for stomach problems, while *C. limonum* L. was used for flu and *P. major* for sore throat. Gastrointestinal tract-related problems were among the main indications for using the eight most utilized medicinal plants. This is relevant since the gastrointestinal tract could be a target of coronaviruses, as they cause both respiratory and gastrointestinal infections in humans and animals^11^.

An overview of 102 systematic reviews identified that the most prevalent respiratory symptoms related to COVID-19 were cough (53.6%), expectoration (23.4%), dyspnea (19.8%), and sore throat (12.4%). Concerning the neurological system, the most frequent symptoms included fever (64.6%), fatigue (29.4%), headache (11.1%), and muscle soreness (18.7%). Gastrointestinal symptoms associated with COVID-19 encompassed anorexia (12.9%), diarrhea (8.1%), nausea (6.7%), vomiting (5.5%), and constipation (5.5%)^12^.

In this regard, we could observe that five of the 10 most commonly used medicinal plants had COVID-19-like symptoms, including indications for headaches, body pain, cough, and unspecified flu-like symptoms. Furthermore, during the pandemic period, an increase in self-medication with drugs to treat symptoms similar to those of COVID-19 was observed in Brazil. The most self-administered drugs included analgesics, antipyretics, muscle relaxants, anti-inflammatories, and anti-flu drugs^13^.

During the COVID-19 pandemic, 63 medicinal plant species used to prevent COVID-19 were investigated and recorded in a Nepal study. The most commonly used species in Nepal, such as *Zingiber officinale*, *Curcuma angustifolia,* and *Allium sativum* L., were the most cited. These species are listed as an alternative medicine to boost people’s immunity by the Nepalese Ministry of Health. Plants such as *C. angustifolia, Cuminum cyminum*, *A. sativum* L., *Terminalia bellirica, Z. officinale, Ocimum tenuiflorum, Cinnamomum species, Piper nigrum, Vitis vinifera,* and *Citrus *spp. have also been recommended by the Indian government to boost immunity but does not claim to cure or treat COVID-19^6^.

In a study conducted in Peru during COVID-19, *Eucalyptus globulus Labill*. was the most commonly used medicinal plant, followed by *Z. officinale Roscoe*, *A. sativum* L., *Piper aduncum* L., *M. recutita* L., and *Erythroxylum coca Lam*^5^. In a Thai study, the medicinal plants most used for the treatment of COVID-19 were *Psidium guajava* L., *followed by Phyllanthus emblica* L., *Houttuynia cordata Thunb*., *P. major* L., and *Strobilanthes cusia (Nees) Kuntze*^14^.

Another study conducted in Morocco identified that 61.9% of the participants used medicinal plants to prevent or treat COVID-19. Among these, 30.6% reported single use since the pandemic’s beginning, 47.8% reported frequent daily use until recovery and 17.4% reported permanent daily single use. *Eucalyptus*, *Syzygium aromaticum*, *Citrus limon*, and *A. sativum* L. are the most used plants^15^.

According to Demeke et al.^16^, in a narrative review, some antiviral medicinal plants such as various species of citrus (*Citrus *spp.), *Citrus sinensis, A. sativum* L*., Allium cepa, Mentha piperita*, and *Nigella sativa* are considered highly beneficial herbal options or fruits. These natural products provide effective supplementary components in managing COVID-19. Furthermore, another study highlighted that combining herbal medicine and Western medicine can significantly improve the clinical symptoms of COVID-19 patients and reduce the duration of treatment for those in a worse clinical state^17^.

Although some medicinal plants mentioned in other studies to treat COVID-19 appear as used in this current study, such as *A. sativum* L., *P. major*, *Z. officinale, and P. guajava,* they were employed for reasons other than COVID-19^5,6^. One hypothesis to be investigated for not adopting medicinal plants to treat COVID-19 in Brazil could be the impact of vaccination on the population, as most of the study population had already been vaccinated with the first and booster doses when the survey was conducted. On the other hand, an increased uptake of over-the-counter medications through self-medication for treating symptoms similar to those of COVID-19 was observed^13^.

Factors associated with using medicinal plants were females, self-declared Black/other, having completed until elementary school, receiving more than two minimum wages, or having comorbidities. This result supports the findings by Patricio et al.^18^, who conducted an integrative review and discovered that women, older adults, and those with low income and schooling were the primary users of medicinal plants in Brazil and worldwide^18^.

It is critical to emphasize that health professionals can help with the rational use of medicinal plants. According to Veiga Junior^19^, medicinal plants were the primary source of treatment for 63% of the population in Rio de Janeiro’s interior. Furthermore, medicinal plants were used as self-medication before consulting a doctor, concurrently with allopathic medicine (55.9%) and, in many cases, replacing it (52.4%) without the doctor’s knowledge.

As implications, it is essential to strengthen the National Program of Medicinal Plants and Herbal Medicines to ensure safe access to and rational use of medicinal plants and herbal medicines for the Brazilian population and promote the sustainable use of its biodiversity. In this regard, educational and informative activities in schools, health services, or communities, and inclusion in academic training curricula for health professionals can contribute to the rational use of medicinal plants, minimizing or preventing cases of poisoning or other harmful effects.

This study has some limitations. The first arises from the study’s nature, which does not allow for establishing a cause-and-effect relationship between observations. There is also a possible memory bias due to using a reminiscent time to identify the use of medicinal plants and describe some explicative variables. Moreover, Brazil exhibits profound regional inequalities, and the study was conducted in the inland territory of the country’s southeastern region. Therefore, the results should be cautiously generalized, especially when considering a population with sociodemographic characteristics different from the present study’s sample.

The study also has important strengths. The first is assessing the consumption of medicinal plants in Brazil during the COVID-19 pandemic. It was a challenging time for many countries, and understanding people’s health behavior during this period is critical for strengthening health actions. Additionally, the study contributes to a widely disseminated and applied integrative and complementary practice in Brazil, which is using medicinal plants and herbal medicines. Thus, these results are expected to contribute to educating the population and health professionals to promote the rational, effective, and safe use of medicinal plants.

In conclusion, 79 different plant species were identified, with no reports of specific use of these plants for the prevention or treatment of COVID-19. Most medicinal plants were employed to relieve anxiety symptoms, which COVID-19 aggravated. Also, medicinal plants used for treating respiratory and gastrointestinal symptoms like those that occur in COVID-19 have been identified. Finally, we identified that women, older adults, Black, Indigenous, or yellow people, the less educated, and those with comorbidities used medicinal plants more frequently.

## Methods

This study is nested in a project designed to perform a situational diagnosis of health during the COVID-19 pandemic in a Brazilian municipality, with the generation of information to assist in health management and pandemic combat. The health profile of the population, the use of medications and medicinal plants, and the utilization of healthcare services were evaluated to achieve this. This specific article focuses on the results related to the use of medicinal plants during the pandemic.

### Study design

A cross-sectional ethnobotanical study was conducted in Alegre, southeastern Brazil. The Federal University of Espírito Santo, campus Alegre, is located in this city, where the research was developed. An on-site household survey was applied in November and December 2021 to conduct the study. According to the census conducted in 2010, Alegre had a population of 30,768 people, of whom 21,512 resided in the urban area of the municipality^20^.

The Consensus-Based Checklist for Reporting Survey Studies (CROSS) was employed as a guideline for reporting this study^21^.

### Population and sampling size

The study population consisted of Alegre residents from urban areas who were at least 18 and consented to participate in the study by signing the Informed Consent Form. The sample size was calculated using the 21,512 urban residents of the municipality as a reference at a 95% confidence level (α error = 0.05), with an expected prevalence of using medicinal herbs of 50%, and the design effect of 1.5. Thus, the minimal sample size was determined to be 567 people, to which 10% was added to account for potential losses, making a total of 624 people who needed to be interviewed^22^.

The World Health Organization-recommended sampling technique with probabilities proportional to size was used to select the subjects to be interviewed^23,24^. For the first stage, 10 out of 37 urban tracts were randomly chosen. In the second stage, a similar number of people should be interviewed in each urban tract. As a result, there was a lower likelihood that a specific person would be sampled^23^ in a larger urban tract.

### Data collection

A pre-coded and structured questionnaire was adopted for data collection (Supplementary Material 2). The questionnaire consisted of a total of 146 questions, subdivided into the following topics: (a) sociodemographic characteristics; (b) general health; (c) COVID-19; (d) use of healthcare services; (e) use of medications; (f) use of teas and medicinal plants; and (g) lifestyle habits. Moreover, the Euro Quality of Life five dimensions three levels questionnaire (EQ-5D-3L) validated in Brazil was employed to measure the quality of life of the respondents^25,26^.

The data collection method was standardized using virtual and presential training. The researchers were submitted to practical training before starting the field work, where information about the data collection instrument and the fieldwork process were reinforced. The questionnaire was adapted from previous research and pre-tested in a sample of 14 people to assess question consistency, respondent comprehension, questionnaire administration time, and the need for adjustments before data collection^27,28^. The technical team responsible for training comprised professors and researchers from the Health Evaluation, Technology, and Economics Group at the Federal University of Espírito Santo (GATES/UFES). In total, 14 researchers participated in the data collection stage.

From then on, data collection was initiated and occurred during the COVID-19 pandemic period. Safety measures were implemented to minimize data collection risks during the pandemic, such as vaccinating the research team (at least two doses), using hand sanitizer during interviews, and wearing face masks and gowns. The researchers walked through the streets of the randomly selected census tracts, and households were approached sequentially without revisiting closed households. One or more individuals could be interviewed in the same household. Additionally, all researchers were accompanied by community health workers who supported the data collection phase.

The use of medicinal plants was investigated for the 15 days preceding the interview, and this variable was obtained from the following question: “Did you drink any tea or use medicinal plants in the last 15 days?”. The name, the portion used (leaf, root, stem, bark, flower, and sachet), and the indication of use for each medicinal plant were identified. The identification of the medicinal plants used, and their respective indications of use was based on the respondent’s self-report, without the researcher’s interference. The intake of fresh products, herbal medicines, and teas was considered as the use of medicinal plants. After self-reporting, the plants mentioned by their common or popular name by respondents were cataloged and identified by their scientific name by two independent researchers, with the support of the online tool Tropicos® version 3.4.2 (www.tropicos.org). The researchers organized the plants according to their family, species, and authority.

A database was constructed after data collection, and the participants’ information was anonymized to ensure information confidentiality.

### Data analysis

Sociodemographic data, such as age, sex, schooling, marital status, ethnicity, and gross monthly income, were first collected, followed by clinical data on diseases, quality of life, health self-perception, sleep hours, physical activity, and alcohol consumption, among other variables.

The frequency distribution was used to assess categorical variables in descriptive data analysis, while the median and interquartile range were adopted to assess continuous variables. The occurrence of missing data was observed in less than 5% of the questionnaires and occurred at random. In this regard, due to the low proportion of missing data and the addition of 10% to the minimum sample size to account for possible data absence, we decided not to perform an imputation of the missing data. Furthermore, the study’s sample size exceeded the estimated minimum sample size. As a result, the analyses were conducted without including the missing data.

Poisson regression with robust variance was used to investigate factors related to medicinal plant use. The explanatory variables of the Poisson model were age, sex, race, marital status, religion, schooling, income, self-perceived health, quality of life by EQ-5D-3L, body mass index classification, regular physical activity, alcohol consumption, smoking, daily sleep hours, medical appointments in the previous year, hospitalizations in the previous year, coverage by a private health plan, use of the pharmaceutical services. Only variables with a p-value < 0.05 remained in the final multivariable model. All data were analyzed using the Jamovi Software version 2.2.5 and Stata version 16.1.

### Ethic consent

The Federal University of Espirito Santo’s Research Ethics Committee approved the study under Opinion N° 4.732.878. The informed consent term was formally approved and signed by all respondents. All procedures followed the human research committee’s ethical standards and the 1964 Helsinki Declaration, revised in 2013^29^.

### Supplementary Information

Supplementary Information.

## Data Availability

The datasets generated and analyzed during the current study are available from the corresponding author upon reasonable request.
