# Supplementary material for: Use of medicinal plants during COVID-19 pandemic in Brazil
Source: Sci Rep. 2023 Oct 2;13:16558. doi: 10.1038/s41598-023-43673-y (PMC10545667; doi:10.1038/s41598-023-43673-y)
Supplement: Supplementary file 1 — Supplementary Information. [file 41598_2023_43673_MOESM1_ESM.pdf]

Scientific Reports - Use of medicinal plants during COVID-19 pandemic in Brazil: findings and implications

Alciellen Mendes da Silva[1], Ana Luísa Horsth[1], Élida Silva Timóteo[1], Ronaldo José Faria[1,2], Patrícia Silva Bazoni[1,2], Eduardo Frizzera Meira[1], Jéssica Barreto Ribeiro dos Santos[1], Michael Ruberson Ribeiro da Silva[1,2]

SUPPLEMENTARY MATERIAL 01 – SUPPLEMENTARY TABLE

Supplementary Table 1 – Medicinal plants used and their use by the study population

**Plant**	**n (%)[1]**	**n (%)[2]**	**Main cited indications (n, %)**
Melissa officinalis L.	80 (11.5)	80 (31.0)	Anxiety (59; 73.8%), hypertension (7; 8.8%), like tea (3; 3.8%), stomach problems (3; 3.8%), insomnia (2; 2.5%), flu (2; 2.5%), liver health (1; 1.2%), replacing coffee (1; 1.2%), immunity (1; 1.2%), no indication (1; 1.2%)
Peumus boldus Molina	63 (9.1)	63 (24.4)	Stomach problems (41; 65.1%), indigestion (8; 12.7%), pain (5; 7.9%), anti-inflammatory (2; 3.2%), nausea (2; 3.2 %), cough (1; 1.6%), dyslipidemia (1; 1.6%), no indication (1; 1.6%), flatulence (1; 1.6%), liver health (1; 1.6%)
Mentha spicata L.	54 (7.8)	54 (20.9)	Anxiety (16; 29.6%), pain (7; 13.0%), stomach problems (5; 9.2%), indigestion (4; 7.4%), no indication (4; 7.4%), like tea (3; 5.6%, gases (2; 3.7%), intestines (2; 3.7%), flu (2; 3.7%), diuretic (2; 3.7%), gastritis (1; 1.8%), hypertension (1; 1.8%), memory (1; 1.8%), parasitosis (1; 1.8%), kidney problems (1; 1.8%), rheumatism (1; 1.8%),
Matricaria recutita L.	47 (6.8)	47 (18.2)	Anxiety (32; 68.0%), like tea (5; 10.6%). Stomach problems (4; 8.5%). Insomnia (3; 6.4%) no indication (1; 2.1%), cramps (1; 2.1%), replacing coffee (1; 2.1%)
Rosmarinus officinalis L.	44 (6.3)	44 (17.0)	Anxiety (26; 59.1%), diuretic (9; 20.4%), indigestion (3; 6.8%), like tea (2; 4.5%), dyslipidemia (1; 2.3%), anti-inflammatory (1; 2.3%), shortness of breath (1; 2.3%), replacing coffee (1; 2.3%)
Pimpinella anisum L.	38 (5.5)	38 (14.7)	Anxiety (20; 52.6%), like tea (4; 10.5%), diuretic (4; 10.5%), indigestion (3; 7.9%), gas (3; 7.9%), replacing coffee (2; 5.3%), insomnia (1; 2.6%), gastritis (1; 2.6%)
Leonurus sibiricus L.	26 (3.7)	26 (10.0)	Stomach problems (11; 42.3%), preventing heart attack (4; 15.4%), headache (3; 11.5%), avoiding stroke (2; 7.7%), like tea (1; 3.8%), inflammation in the tooth (1; 3.8%), reduce symptoms due to stroke (1; 3.8%), hypertension (1; 3.8%), purify the blood (1; 3.8%), liver health (1; 3.8%)
Citrus limonum	25 (3.6)	25 (9.7)	Flu (13; 52.0%), immunity (4; 16.0%), cough (4; 16.0%), lung (1; 4.0%), cold (1; 4.0%), diuretic (1; 4.0%), like tea (1; 4.0%)
Plantago major L.	16 (2.3)	16 (6.2)	Treat infections (7; 43.8%), sore throat (5; 31.2%), antibiotic (2; 12.5 %), inflammation (1; 6.2%), flu (1; 6.2%)
Ilex paraguariensis	13 (1.8)	13 (4.9)	Anxiety (4; 30.8%), replacing coffee (4; 30.8%), like tea (2; 15.4%), weight loss (1; 7.7%), diabetes mellitus (1; 7.7%), diuretic (1; 7.7%)
Rosa alba L.	12 (1.7)	12 (4.6)	Urinary tract infection (4; 33.3%), uterine infection (2; 16.7%), kidney health (2; 16.7%), stomach problems (2; 16.7%), thrush (1; 8.3%), anti-inflammatory (1; 8.3%)
Solidago chilensis	11 (1.6)	11 (4.3)	Pain (5; 45.4%), malaise (2; 18.2%), insomnia (1; 9.1%), flu (1;

			9.1%), bruises (1; 9.1%), lung health (1; 9.1%)
Cymbopogon citratus	8 (1.2)	8 (3.1)	Anxiety (6; 75.0%), insomnia (1; 12.5%), like tea (1; 12.5%)
Equisetum arvense L.	8 (1.2)	8 (3.1)	Diuretic (4; 50.0%), anti-inflammatory (2; 25.0%), cough (1; 12.5%), slimming (1; 12.5%)
Camellia sinensis	8 (1.2)	8 (3.1)	Weight loss (2; 25.0%), diuretic (2; 25.0%), no indication (2; 25.0%), fat in the liver (1; 12.5%), stomach problems (1; 12.5%)
Morus nigra L.	7 (1.0)	7 (2.7)	Menopause (4; 57.1%), polycystic ovaries (1; 14.3%), diabetes mellitus (1; 14.3%), weight loss (1; 14.3%)
Hibiscus rosa-sinensis L.	6 (0.9)	6 (2.3)	Diuretic (4; 66.7%), slimming (2; 33.3%)
Mangifera indica L.	5 (0.7)	5 (1.9)	Flu (3; 60.0%), diabetes mellitus (1; 20.0%), cough (1; 20.0%)
Pereskia aculeata	5 (0.7)	5 (1.9)	No indication (4; 80.0%) and diabetes mellitus (1; 20.0%)
Mentha viridis	4 (0.6)	4 (1.5)	Flu (1; 25.0%), pimple (1; 25.0%), like tea (1; 25.0%), stomach problems (1; 25.0%)
Ananas comosus	3 (0.4)	3 (1.1)	Diuretic (2; 66.7%) and flu (1; 33.3%)
Cinnamomum verum	3 (0.4)	3 (1.1)	Rhinitis (1; 33.3%), muscle pain (1; 33.3%), like tea (1; 33.3%)
Baccharis trimera	3 (0.4)	3 (1.1)	Dyslipidemia (1; 33.3%), hypertension and diabetes mellitus (1; 33.3%), anti-inflammatory (1; 33.3%)
Psidium guajava L.	3 (0.4)	3 (1.1)	Avoid hair loss (1; 33.3%), no indication (1; 33.3%), discomfort (1; 33.3%)
Artemisia absinthium	3 (0.4)	3 (1.1)	Stomach problems (2; 66.7%), abdominal pain (1; 33.3%)
Carica papaya L.	3 (0.4)	3 (1.1)	Cough (1; 33.3%), diabetes mellitus (1; 33.3%), flu (1; 33.3%)
Petroselinum crispum	3 (0.4)	3 (1.1)	Diuretic (2; 66.66%), kidney health (1; 33.33%)
Ocimum basilicum L.	6 (0,9)	6 (2,3)	Hypertension (1; 33.3%), no indication (1; 33.3%), flu (1; 33.3%)
Salvia officinalis L.	3 (0.4)	3 (1.1)	Indigestion (1; 33.3%), labyrinthitis (1; 33.3%), diuretic (1; 33.3%)
Senna alexandrina	3 (0.4)	3 (1.1)	Constipation (2; 66.6%), hypertension and diabetes mellitus (1; 33.3%)
Persea americana	2 (0.3)	2 (0.8)	Urinary tract infection (2; 100.0%)
Schinus terebinthifolia	2 (0.3)	2 (0.8)	Diuretic (1; 50.0%) and infection (1; 50.0%)
Butia eriospatha	2 (0.3)	2 (0.8)	Stomach problems and diarrhea (1; 50.0%), diuretic (1; 50.0%)
Coffea sp.	2 (0.3)	2 (0.8)	Diabetes mellitus and dyslipidemia (1; 50.0%), dizziness (1; 50.0%)
Costus spicatus	2 (0.3)	2 (0.8)	Kidneys and liver (1; 50.0%), anti-inflammatory (1; 50.0%)
Echinodorus grandiflorus	2 (0.3)	2 (0.8)	Kidneys, diuretic, and infection (1; 50.0%), sore throat (1; 50.0%)
Foeniculum vulgare L.	2 (0.3)	2 (0.8)	Anxiety (1; 50.0%), increase breast milk (1; 50.0%)
Zingiber officinale	2 (0.3)	2 (0.8)	Infection and flu (1; 50.0%), sore throat (1; 50.0%)
Citrus sinensis	2 (0.3)	2 (0.8)	Anxiety (1; 50.0%), flu (1; 50.0%)
Erythrina verna	2 (0.3)	2 (0.8)	Anxiety (2; 100.0%)
Cucumis melo L.	2 (0.3)	2 (0.8)	Diabetes mellitus (1; 50.0%), hypothyroidism (1; 50.0%)
Poa annua L.	2 (0.3)	2 (0.8)	Antibiotic (1; 50.0%), lung health (1; 50.0%)
Bidens alba	2 (0.3)	2 (0.8)	Pain (1; 50.0%), nausea (1; 50.0%)
Kalanchoe brasiliensis Cambess	2 (0.3)	2 (0.8)	Expectorant (1; 50.0%), pain (1; 50.0%)
Lepidium meyenii	2 (0.3)	2 (0.8)	Anti-inflammatory (1; 50.0%), no indication (1; 50.0%)
*30 herbs**	1 (0.1)	1 (0.4)	Weight loss (1; 100.0%)
Vernonia polysphaera	1 (0.1)	1 (0.4)	Lung health (1; 100.0%)
Curcuma longa L.	1 (0.1)	1 (0.4)	No indication (1; 100.0%)
Gossypium herbaceum L.	1 (0.1)	1 (0.4)	Urinary tract infection (1; 100.0%)
Allium sativum L.	1 (0.1)	1 (0.4)	Cough (1; 100.0%)
Anadenanthera macrocarpa	1 (0.1)	1 (0.4)	Cough and bronchitis (1; 100.0%)

Aloe vera	1 (0.1)	1 (0.4)	Urinary tract infection (1; 100.0%)
Psidium guajava L.	1 (0.1)	1 (0.4)	No indication (1; 100.0%)
Erythroxylum vaccinifolium	1 (0.1)	1 (0.4)	Cramp (1; 100.0%)
Sechium edule	1 (0.1)	1 (0.4)	Hypertension (1; 100.0%)
Alpinia zerumbet	1 (0.1)	1 (0.4)	Hypertension (1; 100.0%)
*tonic compound**	1 (0.1)	1 (0.4)	Energetic (1; 100.0%)
Syzygium aromaticum	1 (0.1)	1 (0.4)	Like tea (1; 100.0%)
Taraxacum officinale L.	1 (0.1)	1 (0.4)	Hypertension (1; 100.0%)
Cecropia	1 (0.1)	1 (0.4)	Hypertension (1; 100.0%)
Cordia verbenacea DC.	1 0.1)	1 (0.4)	Inflammation (1; 100.0%)
Dysphania ambrosioides	1 (0.1)	1 (0.4)	Parasitosis (1; 100.0%)
Hypericum perforatum L.	1 (0.1)	1 (0.4)	Muscle pain (1; 100.0%)
Maytenus illicifolia	1 (0.1)	1 (0.4)	Dyslipidemia (1; 100.0%)
Cajanus cajan	1 (0.1)	1 (0.4)	Infection and fever (1; 100.0%)
Mikania glomerata	1 (0.1)	1 (0.4)	Flu (1; 100.0%)
Paullinia cupana	1 (0.1)	1 (0.4)	Diuretic and infection (1; 100.0%)
Annona muricata L.	1 (0.1)	1 (0.4)	Diabetes mellitus (1; 100.0%)
Achyrocline satureioides DC.	1 (0.1)	1 (0.4)	Anxiety (1; 100.0%)
Passiflora edulis	1 (0.1)	1 (0.4)	Anxiety (1; 100.0%)
Lippia alba	1 (0.1)	1 (0.4)	Anxiety (1; 100.0%)
Citrus reticulata	1 (0.1)	1 (0.4)	Flu (1; 100.0%)
Morinda citrifolia L.	1 (0.1)	1 (0.4)	Hypertension and dyslipidemia (1; 100.0%)
Oil europaea L.	1 (0.1)	1 (0.4)	Hypertension (1; 100.0%)
Solanum cernuum	1 (0.1)	1 (0.4)	Kidney problems (1; 100.0%)
Quassia amara L.	1 (0.1)	1 (0.4)	Diabetes mellitus (1; 100.0%)
Cucumis sativus L.	1 (0.1)	1 (0.4)	Hypothyroidism (1; 100.0%)
Eugenia uniflora L.	1 (0.1)	1 (0.4)	Diabetes mellitus (1; 100.0%)
Apis mellifera L.	1 (0.1)	1 (0.4)	Sore throat (1; 100.0%)
Mentha pulegium L.	1 (0.1)	1 (0.4)	Bronchitis (1; 100.0%)
Imperata brasiliensis	1 (0.1)	1 (0.4)	Joint pain (1; 100.0%)
Cuphea carthagenensis	1 (0.1)	1 (0.4)	Hypertension (1; 100.0%)

(%)[1] = in relation to the total study population (n=641)
(%)[2] = in relation to people who used medicinal plants (n=242)
* Association of herbal plants.

SUPPLEMENTARY MATERIAL 02 – DATA COLLECTION QUESTIONNAIRE

**PART A - SOCIODEMOGRAPHIC DATA**	
**Name:**	
**Email:**	**Telephone:**
**Neighborhood/District:**	**Mother's name:**
**Naturalness:**	**Questionnaire number:**

**1- Sex:** 1. Female 2. Male	|____|
**2- Date of birth:** ______/______/______ or _________ years	
**3- What is your weight?**<br>_________ Kilograms	|____|
**4- How tall are you?**<br>_________ centimeters or _________ metre	|____|
**5- Race or color:**<br>1. White 4. Yellow 7. Other:<br>2. Black 5. Indigenous Specify:______________________<br>3. Brown 6. Albino	|____|
**6- Marital status:**<br>1. Single 4. Divorced 7. Other<br>2. Married 5. Separate Specify:______________________<br>3. Stable Union 6. Widower	|____|
**7- Education:** ________ full years of study<br>1. No schooling 6. Complete technician<br>2. Incomplete elementary school (1st grade) 7. Complete Superior<br>3. Complete elementary school (1st grade) 8. Complete Master's Degree<br>4. Incomplete high school (2nd degree) 9. Full PhD<br>5. Complete high school (2nd degree)	|____|
**8- You live in:**<br>1. Own house or apartment (of family or friends)<br>2. Rented house or apartment<br>3. Bedroom, room, or kitchenette rented or borrowed<br>4. Republic<br>If another, (hotel, asylum, etc.) specifies: ____________________________________	|____|
**9- Religion/Belief**<br>1. No religion 3. Evangelical/Protestant 5. Other<br>2. Catholic 4. Spiritist<br>Specify: ____	|____|
**10- Do you live temporarily in Alegre for study or work?**<br>1. Yes, study 2. Yes, work 3. No	|____|
**11- Lives with**<br>1. Alone 3. Relatives (grandparents/uncles)<br>2. Family members (Parents and/or 4. Friends	|____|
**12- Occupation**<br>1. Self-employed/Professional/Own account<br>2. Employer/Entrepreneur /Entrepreneur<br>3. Formal private employee (CLT or contract)<br>4. Civil servant (Public companies, e.g. bank of Brazil, cashier, etc.)<br>5. Public servant<br>6. Student<br>7. Other. Specify:____	|____|
**13- What is your profession?** ____<br>____	

**14- Gross monthly income in minimum wages:**<br>1. ≤ 1 Salary (< R$ 1,100)<br>2. 1 to 2 Salaries (R$ 1.101 - R$ 2.200)<br>3. 2 to 4 Salaries (R$ 2.201 - R$ 4.400)<br>4. 4 to 10 salaries (R$ 4.401 - R$ 11.000)<br>5. > 10 Salaries (> R$ 11,001)	⌊___⌋
**PART B - GENERAL HEALTH AND USE OF HEALTH SERVICES**	
**15- Generally speaking , you consider your own state of health as:**<br>1. Very good 3. Regular 5. Too bad<br>2. Good 4. Bad	⌊___⌋
**16- In the last 15 days, you have stopped doing some of your usual activities (e.g. leaving home, going for a walk or working) for any health problems?**<br>1. Yes 2. No	⌊___⌋
**17- In the last 15 days, have you been bedridden for any health problems?**<br>1. Yes 2. No	⌊___⌋
**18- From 1 year to now, how many times have you consulted with a doctor?**<br>0 1 2 3 4 5 >5	⌊___⌋
**19- From 1 year to now, how many times have you consulted with a dentist?**<br>0 1 2 3 4 5 >5	⌊___⌋
**20- From 1 year to now, how many times have you consulted with a nutritionist?**<br>0 1 2 3 4 5 >5	⌊___⌋
**21- From 1 year to now, how many times have you consulted with another professional?**<br>0 1 2 3 4 5 >5<br>Which:____	⌊___⌋
**22- From 1 year to now, how many times have you been admitted to a hospital? Consider hospitalization when you have slept in the hospital for at least one night for health problem or accident with you.**<br>1. Not once 2. Once 3. Two or more	⌊___⌋
**23- In addition to the care provided by SUS, do you have private health insurance?**<br>1. Yes 2. No	⌊___⌋
**24- Does your private health plan pay or provide medicines for you?**<br>1. Yes 2. No 3. You don't have private health insurance	⌊___⌋
**25- What is the main problem when you need health services?**<br>1. It's hard to get to the point of care (no transportation or away from home)<br>2. It's hard to make a doctor's appointment (have to stand in line)<br>3. The price of services is very expensive<br>4. It's hard to get a doctor who attends at the time you need it<br>5. Low quality of care<br>6. Lack of medicines and supplies<br>7. You don't have difficulties when you need medical services<br>8. You don't need medical services<br>9. If other, specify: ____	⌊___⌋
**Have you ever in your life a doctor or other health care professional said you have or have you had any of the following diseases? (If necessary, check more options.)**<br>1. Yes 2. No	
**26- Asthma**	⌊___⌋
**27- Anxiety**	⌊___⌋
**28- Rheumatoid arthritis**	⌊___⌋
**29- Cancer / Tumor / Neoplasms**	⌊___⌋
**30- Depression**	⌊___⌋
**31- Diabetes Mellitus**	⌊___⌋
**32- Dyslipidemia (high cholesterol and /or triglycerides)**	⌊___⌋
**33- Heart diseases (heart attack, heart failure, angina, etc.)**	⌊___⌋

**34- Gastroesophageal reflux disease**<br>**35- Kidney diseases of any nature**<br>**36- Hypertension (high blood pressure)**<br>**37- Obesity (overweight)**<br>**38- Hypothyroidism**<br>**39- Other, please specify:** ___	|__| |__| |__| |__| |__| |__|
**40- Has a disability:**<br>1. Yes 2. No	|__|
**41- What type of disability**<br>1. Hearing 3. Cognitive 5. Other<br>2. Visual 4. Physics<br>**Especificar:** ___	|__|
**PART C – COVID-19**	
**42- Got vaccinated?**<br>1. Yes 2. No	|__|
**43- Which vaccine?**<br>1. Coronavac 3. Pfizer 5. Other<br>2. Astra-zeneca 4. Jannsen	|__|
**44- How many doses?**<br>1. One serving 2. Two doses 3. Three doses 4. Single dose	|__|
**45- Have you had an allergic reaction to the vaccine?**<br>1. Yes 2. No	|__|
**46- If so, which one?**<br>Specify: ___	|__|
**47- Did you have COVID-19?**<br>1. Yes 2. No	|__|
**48- When?**<br>1. Before vaccination 3. After the second dose<br>2. After the first dose	|__|
**49- Have you received any medication or treatment?**<br>1. Yes 2. No	|__|
**50- If yes, was it prescribed or indicated by any professional?**<br>1. Yes, medical prescription 4. Yes, referral from friends or relatives<br>2. Yes, pharmaceutical indication 5. No, you searched the internet<br>3. Yes, referral from another professional 6. No, he took it on his own	|__|
**51- If so, what medications were used?**<br>Specify: ___	
**52- Were you hospitalized?**<br>1. Yes 2. No<br>Time in days: ___	|__|
**PART D – USE OF HEALTH SERVICES**	
**53- 53- In the last year you used the Municipal Basic Pharmacy?**<br>1. Yes 2. No	|__|
**54- If yes, what is your level of satisfaction with the service offered?**<br>Totally Unsatisfied 0 ___ 10 Totally Satisfied	|__|
**55- In the last year you used the Emergency Room / Municipal Emergency Room?**<br>1. Yes 2. No	|__|
**56- If so, what is your level of satisfaction with the service offered?**<br>Totally Unsatisfied 0 ___ 10 Totally Satisfied	|__|

**57- In the last year you used the Municipal Medical Center?**<br>1. Yes 2. No	|___|
**58- If so, what is your level of satisfaction with the service offered?**<br>Totally Unsatisfied 0 ____________________ 10 Totally Satisfied	|___|
**59- In the last year did you use the Basic Health Unit?**<br>1. Yes 2. No<br>If yes, specify which: ____	|___|
**60- If so, what is your level of satisfaction with the service offered?**<br>Totally Unsatisfied 0 ____________________ 10 Totally Satisfied	|___|
**61- In the last year you used the Hospital of Alegre?**<br>1. Sim 2. Não	|___|
**62- If so, what is your level of satisfaction with the service offered?**<br>Totally Unsatisfied 0 ____________________ 10 Totally Satisfied	|___|
**63- In the last year you used the Immunization Center of Alegre?**<br>1. Sim 2. Não	|___|
**64- If so, what is your level of satisfaction with the service offered?**<br>Totally Unsatisfied 0 ____________________ 10 Totally Satisfied	|___|
**65- In the last year did you use the Psychosocial Care Center of Alegre?**<br>1. Sim 2. Não	|___|
**66- If so, what is your level of satisfaction with the service offered?**<br>Totally Unsatisfied 0 ____________________ 10 Totally Satisfied	|___|
**67- In the last year have you had any private medical consultation in Alegre?**<br>1. Sim 2. Não	|___|
**68- If so, what is your level of satisfaction with the service offered?**<br>Totally Unsatisfied 0 ____________________ 10 Totally Satisfied	|___|
**69- In the last year you used any Private Pharmacy in Alegre?**<br>1. Sim 2. Não	|___|
**70- If so, what is your level of satisfaction with the service offered?**<br>Totally Unsatisfied 0 ____________________ 10 Totally Satisfied	|___|
**PART E - USE OF MEDICINES**	
In the next questions, we want to know some information about the use of medications by you.<br>Note: If you do not use medicines, skip to question no. 121.	
**71- In the last 15 days, have you used medications?**<br>1. Yes. How many?________ 2. No 99. NSA	|___|
**72- Do you need help from someone else to take your medications?**<br>1. No 2. Yes, sometimes 3. Yes, always	|___|
**73- How many dollars have you spent on medicines in the last 30 days?**<br>R$_____,____ reais<br>1. I didn't spend anything 2. I don't know the value	|___|___|___|
**74- Do you usually use any medications on your own?**<br>1. Yes 2. No 99. NSA	|___|
**75- In the last 15 days have you stopped using any prescription drugs?**<br>1. Yes 2. No	|___|
**MEDICATION 1**	
**(copy this information preferably from the packaging or prescription)**	

**76- Name:**<br>____	
**77- Dosage:**<br>____	|___|
**78- Frequency of use:**<br>____	|___|
**79- How long have you been using this medicine?**<br>1. Less than 1 year 2. 1 year or more	|___|
**80- Where has this medicine been prescribed or recommended?**<br>1. In consultation with the doctor 4. On the radio/TV/newspaper<br>2. In consultation with the dentist 5. Friends or relatives or neighbors<br>3. In the pharmacy 6. Use on your own	|___|
**81- Where did you last get this medicine?**<br>1. In the SUS or public pharmacy 3. Other<br>2. In the commercial pharmacy<br>Specify: ____	|___|
**82- Do you have any problems getting or using this medicine?**	
1. Nope. No problem 4. Yes. There's no one to look for<br>2. Yes. Not in the SUS pharmacy 5. Yes. You don't have money to buy<br>3. Yes. I got sick while using the medicine 6. Yes. Another problem<br>Specify: ______________________________	|___|
**83- Have you stopped taking this medication for some reason in the last 15 days?**<br>1. Yes 2. No	|___|
**84- Why did you stop taking this medicine in the last 15 days.**<br>____	|___|
**MEDICATION 2**	
**(copy this information preferably from the packaging or prescription)**	
**85- Name:**<br>____	|___|
**86- Dosage:**<br>____	|___|
**87- Frequency of use:**<br>____	|___|
**88- How long have you been using this medicine?**<br>1. Less than 1 year 2. 1 year or more	|___|
**89- Where has this medicine been prescribed or recommended?**<br>1. In consultation with the doctor 4. On the radio/TV/newspaper<br>2. In consultation with the dentist 5. Friends or relatives or neighbors<br>3. In the pharmacy 6. Use on your own	|___|
**90- Where did you last get this medicine?**<br>1. In the SUS or public pharmacy 3. Other<br>2. In the commercial pharmacy<br>Specify: ____	|___|
**91- Do you have any problems getting or using this medicine?**	
1. Nope. No problem 4. Yes. There's no one to look for<br>2. Yes. Not in the SUS pharmacy 5. Yes. You don't have money to buy<br>3. Yes. I got sick while using the medicine 6. Yes. Another problem<br>Specify: ______________________________	|___|
**92- Have you stopped taking this medication for some reason in the last 15 days?**<br>1. Yes 2. No	|___|
**93- Why did you stop taking this medicine in the last 15 days.**<br>____	|___|

MEDICATION 3	
(copy this information preferably from the packaging or prescription)	
94- Name: ______________________________	|___|
95- Dosage: ______________________________	|___|
96- Frequency of use: ______________________________	|___|
97- How long have you been using this medicine?<br>1. Less than 1 year 2. 1 year or more	|___|
98- Where has this medicine been prescribed or recommended?<br>1. In consultation with the doctor 4. On the radio/TV/newspaper<br>2. In consultation with the dentist 5. Friends or relatives or neighbors<br>3. In the pharmacy 6. Use on your own	|___|
99- Where did you last get this medicine?<br>1. In the SUS or public pharmacy 3. Other<br>2. In the commercial pharmacy<br>Specify: ______________________________	|___|
100- Do you have any problems getting or using this medicine?	
1. Nope. No problem 4. Yes. There's no one to look for<br>2. Yes. Not in the SUS pharmacy 5. Yes. You don't have money to buy<br>3. Yes. I got sick while using the medicine 6. Yes. Another problem<br>Specify: ______________________________	|___|
101- Have you stopped taking this medication for some reason in the last 15 days?<br>1. Yes 2. No	|___|
102- Why did you stop taking this medicine in the last 15 days. ______________________________	|___|
MEDICATION 4	
(copy this information preferably from the packaging or prescription)	
103- Name: ______________________________	|___|
104- Dosage: ______________________________	|___|
105- Frequency of use: ______________________________	|___|
106- How long have you been using this medicine?<br>1. Less than 1 year 2. 1 year or more	|___|
107- Where has this medicine been prescribed or recommended?<br>1. In consultation with the doctor 4. On the radio/TV/newspaper<br>2. In consultation with the dentist 5. Friends or relatives or neighbors<br>3. In the pharmacy 6. Use on your own	|___|
108- Where did you last get this medicine?<br>1. In the SUS or public pharmacy 3. Other<br>2. In the commercial pharmacy<br>Specify: ______________________________	|___|
109- Do you have any problems getting or using this medicine?<br>1. Nope. No problem 4. Yes. There's no one to look for<br>2. Yes. Not in the SUS pharmacy 5. Yes. You don't have money to buy<br>3. Yes. I got sick while using the medicine 6. Yes. Another problem<br>Specify: ______________________________	|___|
110- Have you stopped taking this medication for some reason in the last 15 days?<br>1. Yes 2. No	|___|

**111- Why did you stop taking this medicine in the last 15 days.**<br>____	|____|
**MEDICATION 5**	
**(copy this information preferably from the packaging or prescription)**	
**112- Name:**<br>____	|____|
**113- Dosage:**<br>____	|____|
**114- Frequency of use:**<br>____	|____|
**115- How long have you been using this medicine?**<br>1. Less than 1 year 2. 1 year or more	|____|
**116- Where has this medicine been prescribed or recommended?**<br>1. In consultation with the doctor 4. On the radio/TV/newspaper<br>2. In consultation with the dentist 5. Friends or relatives or neighbors<br>3. In the pharmacy 6. Use on your own	|____|
**117- Where did you last get this medicine?**<br>1. In the SUS or public pharmacy 3. Other<br>2. In the commercial pharmacy<br>Specify: ____	|____|
**118- Do you have any problems getting or using this medicine?**	
1. Nope. No problem 4. Yes. There's no one to look for<br>2. Yes. Not in the SUS pharmacy 5. Yes. You don't have money to buy<br>3. Yes. I got sick while using the medicine 6. Yes. Another problem<br>Specify: ________________________________	|____|
**119- Have you stopped taking this medication for some reason in the last 15 days?**<br>1. Yes 2. No	|____|
**120- Why did you stop taking this medicine in the last 15 days.**<br>____	|____|
**PART F - USE OF TEAS AND MEDICINAL PLANTS**	
In the next questions, we want to know some information about the use of teas and medicinal plants.<br>Note: If you do not use teas or medicinal plants, skip to question no. 132.	
**121- Have you had any tea or used a medicinal plant in the last 15 days?**<br>1. Yes 2. No 99. NSA	|____|
**PLANT 1**	
**122- Name:** ____<br>**Indication:** ____	
**123- Part used:**<br>1. Leaf 3. Stalk 5. Flower<br>2. Root 4. Bark 6. Sachet 99. NSA	|____|
**PLANT 2**	
**124- Name:** ____<br>**Indication:** ____	|____|
**125- Part used:**<br>1. Leaf 3. Stalk 5. Flower<br>2. Root 4. Bark 6. Sachet 99. NSA	|____|
**PLANT 3**	
**126- Name:** ____<br>**Indication:** ____	
**127- Part used:**<br>1. Leaf 3. Stalk 5. Flower<br>2. Root 4. Bark 6. Sachet 99. NSA	|____|

**PLANT 4**	
**128- Name:** ________________________________<br>**Indication:** ________________________________	
**129- Part used:**<br>1. Leaf 3. Stalk 5. Flower<br>2. Root 4. Bark 6. Sachet 99. NSA	|___|
**PLANT 5**	
**130- Name:** ________________________________<br>**Indication:** ________________________________	
**131- Part used:**<br>1. Leaf 3. Stalk 5. Flower<br>2. Root 4. Bark 6. Sachet 99. NSA	|___|
**PART G - USE OF HOMEOPATHIC, HERBAL MEDICINES, MANIPULATED AND HOMEMADE**	
**132- Do you make use of homeopathic medicines?**<br>1. Yes 2. No<br>Indication: ________________________________	|___|
**133- Does it make use of Bach's Floral?**<br>1. Yes 2. No<br>Indicação: ________________________________	|___|
**134- Do you use herbal medicines?**<br>1. Yes 2. No<br>Indication: ________________________________	|___|
**135- Do you use manipulated medications?**<br>1. Yes 2. No<br>Indication: ________________________________	|___|
**136- Use of home medicines?**<br>1. Yes 2. No<br>Specify: ________________________________<br>Indication: ________________________________	|___|
**PART H - LIFESTYLE HABITS**	
**Think about your weekly routine: what are the meals you usually eat on the day?**<br>1. Yes 2. No<br>**137- Breakfast**<br>**138- Morning snack**<br>**139- Lunch**<br>**140- Snack or afternoon coffee**<br>**141- Dinner or evening coffee**<br>**142- LSnack before bed (supper)**	 <br>|___| |___| |___| |___| |___| |___|
**143- How often do you drink alcohol?**<br>1. I never drink 3. Weekly<br>2. Daily 4. Monthly	|___|
**144- You do REGULAR physical activity, that is, at least 30 minutes.**<br>1. Yes 2. No<br>Weekly frequency: ________________________________	|___|
**145- Do you currently smoke?**<br>1. Yes 2. No	|___|
**146- How many hours a day do you usually sleep?**<br>1. < 6 hours 3. 7 to 8 hours<br>2. 6 to 7 hours 4. > 8 hours	|___|

QUESTIONNAIRE_____________________________DATE:_____/___/___

INTERVIEWEE____

EQ-5D-3L

At this time, we want to understand how your current health status is. I will read three statements in each question and you will indicate which one best describes your state of health today.

38A- Mobility

- ❍ 1 - I have no problems in walking about
- ❍ 2 - I have some problems in walking about
- ❍ 3 - I am confined to bed

38B- Self-care

- ❍ 1 - I have no problems with self-care
- ❍ 2 - I have some problems washing or dressing myself
- ❍ 3 - I am unable to wash or dress myself

38C- Usual Activities (e.g. work, study, housework, family or leisure activities)

- ❍ 1 - I have no problems with performing my usual activities
- ❍ 2 - I have some problems with performing my usual activities
- ❍ 3 - I am unable to perform my usual activities

38D- Pain/Discomfort

- ❍ 1 - I have no pain or discomfort
- ❍ 2 - I have moderate pain or discomfort
- ❍ 3 - I have extreme pain or discomfort

38E- Anxiety/Depression

- ❍ 1- I am not anxious or depressed
- ❍ 2 - I am moderately anxious or depressed
- ❍ 3 - I am extremely anxious or depressed

VISUAL ANALOGIC SCALE

To help people say how good or bad their health status is, we have drawn a scale (similar to a thermometer) on which the best health status you can imagine is marked by 100 and the worst health status you can imagine is marked by 0.

We would like you to indicate on this scale how good or bad is, in your opinion, your state of health today. Please draw a line from the square below to the point on the scale that best describes your health status today.

WHAT IS YOUR HEALTH STATUS TODAY?

MAKE A LINE LEAVING THIS FRAME TO THE SCALE ON THE SIDE

The best health
you can imagine

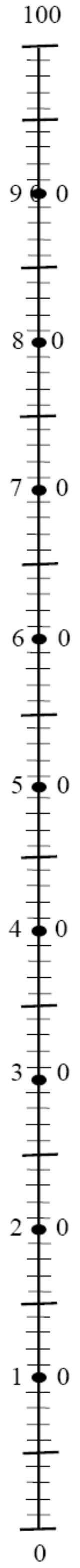

The worst health
you can imagine
